# Long-Term Stimulation with Electroacupuncture at DU20 and ST36 Rescues Hippocampal Neuron through Attenuating Cerebral Blood Flow in Spontaneously Hypertensive Rats

**DOI:** 10.1155/2013/482947

**Published:** 2013-03-31

**Authors:** Gui-Hua Tian, Kai Sun, Ping Huang, Chang-Man Zhou, Hai-Jiang Yao, Ze-Jun Huo, Hui-Feng Hao, Lei Yang, Chun-Shui Pan, Ke He, Jing-Yu Fan, Zhi-Gang Li, Jing-Yan Han

**Affiliations:** ^1^School of Acupuncture and Moxibustion, Beijing University of Chinese Medicine, Beijing 100029, China; ^2^Dongzhimen Hospital Affiliated to Beijing University of Chinese Medicine, Beijing 101121, China; ^3^Tasly Microcirculation Research Center, Peking University Health Science Center, Beijing 100191, China; ^4^Key Laboratory of Microcirculation, State Administration of Traditional Chinese Medicine of the People's Republic of China, Beijing 100191, China; ^5^Department of Anatomy, School of Basic Medical Sciences, Peking University, Beijing 100191, China; ^6^Department of Integration of Chinese and Western Medicine, School of Basic Medical Sciences, Peking University, Beijing 100191, China

## Abstract

This study was designed to investigate the effect of long-term electroacupuncture at Baihui (DU20) and Zusanli (ST36) on cerebral microvessels and neurons in CA1 region of hippocampus in spontaneously hypertensive rats (SHR). A total of 45 male Wistar rats and 45 SHR were randomly grouped, with or without electroacupuncture (EA) at DU20 and ST36, once every other day for a period of 8 weeks. The mean arterial pressure (MAP) was measured once every 2 weeks. Cerebral blood flow (CBF) and the number of open microvessels in hippocampal CA1 region were detected by Laser Doppler and immunohistochemistry, respectively. Nissl staining and Western blotting were performed, respectively, to determine hippocampus morphology and proteins that were implicated in the concerning signaling pathways. The results showed that the MAP in SHR increased linearly over the observation period and was significantly reduced following electroacupuncture as compared with sham control SHR rats, while no difference was observed in Wistar rats between EA and sham control. The CBF, learning and memory capacity, and capillary rarefaction of SHR were improved by EA. The upregulation of angiotensin II type I receptor (AT1R), endothelin receptor (ETAR), and endothelin-1 (ET-1) in SHR rats was attenuated by electroacupuncture, suggesting an implication of AT1R, ETAR, and ET-1 pathway in the effect of EA.

## 1. Introduction

The incidence of hypertension is up to about 30% in the world [1]. Severe, long-term hypertension is accompanied by continuous vasoconstriction that has influence on end blood supply and can eventually lead to vital target organ damage such as heart failure, cerebral disease, and renal failure, the diseases known to be related to the microcirculatory alterations [2].

Cognitive impairment is one of the principal cerebrovascular diseases evoked by hypertension. Several epidemiologic studies have indicated a correlation between blood pressure level and cognitive decline or vascular dementia [3–5]. It has been well accepted that a reduction in the number of small arterioles and capillaries per volume of tissue (rarefaction) plays a major role in the elevation of vascular resistance and, consequently, of blood pressure. On the other hand, impairment of cerebral perfusion resulting from rarefaction contributes to hypoperfusion of the brain, leading to neuronal dysfunction and progressive cognitive failure [6, 7]. Therefore, besides lowering of blood pressure, attenuation of rarefaction and resultant hypoperfusion in cerebral tissue is reasoned as an essential goal for the treatment of cognitive dysfunction following hypertension.

Baihui (DU20), an acupoint in humans located on the top of the head at the intersection of middle sagittal line and the connection of two ear apexes, is extensively used in Chinese medicine for management of palpitations, forgetfulness, dementia and insomnia, and so forth [8]. Zusanli (ST36) is located 3 cm below Dubi (ST35) and one finger-breadth before the anterior crest of the tibia, and is known as an acupoint for its role in reducing blood pressure [9]. A recently published study showed that acupuncture at DU20 can up-regulate brain derived neurotrophic factor (BDNF) expression and facilitate the support of BDNF for neurons, thus ameliorate learning and memory in early dementia rats [10]. In clinic, long-term electroacupuncture stimulation at acupoints DU20 and ST36 has an obvious effect on both reducing blood pressure and protection of forgetfulness. However, it is so far unclear whether or not electroacupuncture at both acupoints DU20 and ST36 can reverse cerebral rarefaction and further ameliorate neuronal dysfunction and, if yes, what are the underlying mechanisms. In this study, using spontaneously hypertensive rats (SHR), we demonstrated the recovery effects of long-term electroacupuncture at DU20 and ST36 on cerebral blood flow (CBF) in cortex, and density of small arterioles and capillaries in hippocampal CA1 region, as well as learning and memory capacity, which can be attributed to the suppression of angiotensin II type 1 receptor (AT1R), endothelin-1 (ET-1), and endothelin receptor (ETAR) in brain tissues.

## 2. Materials and Methods

### 2.1. Animals

Male Wistar rats and SHR (8 weeks) were obtained from the Animal Center of Peking University Health Science Center (Beijing, certificate no. SCXK 2006-0008). SHR are a genetic model of hypertension that is widely accepted in medical research because of the features they share with idiopathic hypertension in humans. This model was developed by Okamoto and Aoki in Kyoto School of Medicine, 1963, from outbred Wistar Kyoto male with marked elevation of blood pressure mated to female with slightly elevated blood pressure [11]. The animals were housed in cages at 22 ± 2°C and humidity of 40 ± 5% under a 12-hour light/dark cycle and received standard diet and water ad libitum. The experimental procedures were in accordance with the European commission guidelines (2010/63/EU). All animals were handled according to the guidelines of the Peking University Animal Research Committee. The protocols were approved by the Committee on the Ethics of Animal Experiments of the Health Science Center of Peking University (LA2011-38).

### 2.2. Electroacupuncture Treatment and Animal Grouping

A total of 45 Wistar rats and 45 SHR were randomly divided into 6 groups: Wistar group (*n* = 15), Wistar + Sham group (Wistar rats with stimulation at nonacupoints, *n* = 15), Wistar + EA group (Wistar rats with stimulation at acupoints, *n* = 15), SHR group (*n* = 15), SHR + Sham group (SHR with stimulation at nonacupoints, *n* = 15), and SHR + EA group (SHR with stimulation at acupoints, *n* = 15). The animals in Wistar + EA group and SHR + EA group were subjected to stimulation by electroacupuncture at acupoint DU20 (located at the midmost point of parietal bone) and ST36 (5 mm below head of right fibula under knee joint, and 2 mm lateral to the anterior tubercle of the tibia). Sterilized disposable stainless steel needles (0.3 mm × 40 mm, Global brand, Suzhou, China) were inserted 2 mm deep at DU20 with a slope of 30 degrees. Perpendicular needling was performed with the depth of 5 mm at ST36. Both needles were connected to Han's Acupoint Nerve Stimulator (Model LH 202H, Huawei Ltd, Beijing, China). To keep animals quiet during electrostimulation, the rats were fastened to an animal plate and adapted for 10 min before electroacupuncture stimulation. Electric stimulation proceeded for 20 minutes each time, once every other day, for a period of 8 weeks, and the stimulation parameters were set at disperse-dense waves of 2/100 Hz with an intensity of 1 mA, 2 Hz [12]. In Wistar + Sham group and SHR + Sham group, the animals received similar treatment as electroacupuncture groups but the electroacupuncture site was 1 cm and 2 cm from the root of the tail, respectively, to replace DU20 and ST36 [13]. The animals in Wistar group and SHR group underwent a similar procedure but without electroacupuncture.

### 2.3. Blood Pressure Measurement

Blood pressure was measured as described [14], with modification. The measurement was conducted once every 2 weeks at 8 am in a quiet room. After staying in a box at 29 ± 1°C for 10 min, the mean arterial pressure was measured with a blood pressure monitor (BP-98A, U0130163, Tokyo, Japan), taking the average of three consecutive measurements as the mean arterial pressure (MAP).

### 2.4. Assessment of CBF

 CBF was measured using a laser Doppler perfusion imager (PeriScan PIM3; PERIMED, Stockholm, Sweden) at the end of the observation. For this purpose, an incision was made through the scalp, and the skin was retracted to expose the skull. The periosteal connective tissue adherent to the skull was removed with a sterile cotton swab. A computer-controlled optical scanner directed a low-powered He-Ne laser beam over the exposed cortex. The scanner head was positioned in parallel to the cerebral cortex at a distance of 18.5 cm. The scanning procedure took 4 sec for a measurement covering an area of 80 pixels. At each measuring site, the beam illuminated the tissue to a depth of 0.5 mm. A color-coded image to denote specific relative perfusion levels was displayed on a video monitor.

### 2.5. Morris Water Maze Test

Cognitive function was tested by the water maze at the end of the observation. The Morris water maze test was conducted according to Morris [15]. The water-filled (23 ± 1°C) black-colored tank (150 cm diameter, 60 cm depth) was divided into four quadrants of equal area arbitrarily. A round platform (10 cm in diameter) made of transparent perspex was submerged 1 cm below the water surface with its center 37.5 cm from the perimeter, in the middle of one quadrant (the target quadrant). A closed-circuit television camera was mounted onto the ceiling directly above the center of the pool to convey subject swimming trajectories and parameters to an electronic image analyzer. 

### 2.6. Tissue Preparation for Histology

Animals in each group (*n* = 6) were anesthetized with pentobarbital sodium (0.1 g/kg body weight) intraperitoneally at 16 weeks and perfused transcardially with 0.9% saline followed by 4% paraformaldehyde in 0.1 M PBS (pH 7.4) for 40 min [16]. The brain was removed and cut into blocks, embedded in paraffin, and sectioned at 10 *μ*m.

Three sections of cerebral hippocampus were collected in each animal. The sections were deparaffinized and rehydrated, sequentially, and examined by Nissl staining or immunohistochemistry as detailed below.

### 2.7. Nissl Staining

The sections were stained with cresyl violet and examined with a light microscope (BX512DP70, Olympus, Tokyo, Japan), according to the standard procedure [17]. Five fields of CA1 sector in hippocampus of each animal were randomly selected, and the number of surviving pyramidal cells per 2 mm of CA1 region was counted.

### 2.8. Immunohistochemistry

The sections were incubated with antibody against CD31 (Thermo Scientific, MA1-80069, Waltham, USA) after blocking with bovine serum albumin. The samples were then incubated with a biotinylated secondary antibody followed by avidin-biotin-peroxidase complex. As control, a consecutive section was treated similarly except that the primary antibody was omitted. Positive staining was visualized with diaminobenzidine. The images were captured by a digital camera connected to a microscope (BX512DP70, Olympus, Tokyo, Japan) and analyzed with Image-Pro Plus 5.0 software (IPP, Media Cybernetic, Bethesda, MD, USA). Five fields of CA1 region were examined for each animal.

### 2.9. ELISA

At 16 weeks, animals from each group (*n* = 8) were anesthetized and the hippocampus of brain was removed and homogenized in lysis buffer including protease inhibitor on ice. After being centrifuged at 20000 rcf for 60 minutes, the supernatant was collected for determination of endothelin-1 (ET-1) and NO content in cerebral tissues by ELISA, according to the manufacture's instruction (Abcam, Cambridge, UK).

### 2.10. Western Blot Analysis

Western blot analysis (*n* = 3) was performed as described previously [18]. Briefly, the brain was removed at week 16, and hippocampus was homogenized in lysis buffer including protease inhibitors. One hundred micrograms of the supernatant was mixed with 4× sample buffer. The protein samples were separated on Tris-glycineSDS-PAGE in a reducing condition. The rabbit primary antibodies used included those that directed against AT1R (1 : 300, Abcam, Cambridge, UK), AT2R (1 : 500, Abcam, Cambridge, UK), ETAR (1 : 500, Abcam, Cambridge, UK), eNOS (1 : 1000, BD, Franklin Lakes, USA), iNOS (1 : 200, Abcam, Cambridge, UK), and GAPDH (1 : 2000, Cell Signaling Technology, Boston, MA, USA). After washing with Tris-buffered saline containing 0.05% Tween-20, the membrane was incubated with horseradish peroxidase-conjugated goat anti-rabbit secondary antibody (1 : 3000, Santa Cruz Biotechnology, Santa Cruz, USA) at room temperature for 60 min. The membranes were analyzed using the enhanced chemiluminescence system, according to the manufacturer's protocol and exposed in a dark box. The protein signal was quantized by scanning densitometry in the X-film by bio-image analysis system (Image-Proplus 5.0, Media Cybermetrics, Bethesda, MD, USA). The results from each experimental group were expressed as relative integrated intensity compared with that from the sham group.

### 2.11. Statistical Analysis

All parameters are expressed as means ± SD. For comparison of >2 conditions a one-way analysis of variance (ANOVA) with Turkey post hoc test or a repeated measures ANOVA with Bonferroni post hoc test was used. A probability of less than 0.05 was considered to be statistically significant.

## 3. Results

### 3.1. Long-Term Stimulation with Electroacupuncture Reduces the MAP in SHR

**Figure 1:**
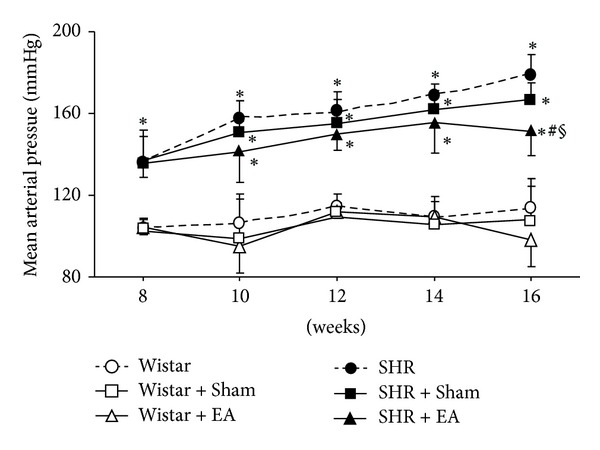
The effect of electroacupuncture on rat MAP. Wistar: Wistar rats without any treatment. Wistar + Sham: Wistar rats with stimulation at nonacupoints. Wistar + EA: Wistar rats with stimulation at acupoints. SHR: SHR without any treatment. SHR + Sham: SHR with stimulation at nonacupoints. SHR + EA: SHR with stimulation at acupoints. Data were expressed as mean ± SD from six animals. *P < 0.05 versus 8 weeks; ^#^ *P* < 0.05 versus SHR group; ^§^ *P* < 0.05 versus SHR + Sham group.

Figure 1 shows the effect of long-term stimulation with electroacupuncture at DU20 and ST36 on MAP during the observation period. MAP in Wistar groups remained nearly unchanged from 8 through 16 weeks. In contrast, MAP in SHR group increased with time, from 130 mmHg at week 8 to 170 mmHg at week 16. MAP in SHR + Sham group changed over time similarly to that in SHR group, and no significant difference was observed at any time point between the two groups. Of notice, MAP in SHR + EA group was attenuated significantly at week 16, as compared to SHR group and SHR + Sham group (*P* < 0.05).

### 3.2. Long-Term Stimulation with Electroacupuncture Increases the CBF in SHR

**Figure 2:**
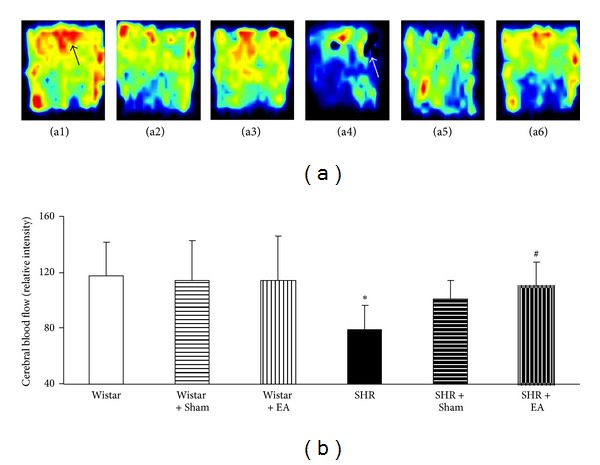
The effect of electroacupuncture on CBF in rat cerebral cortex. (a) Representative laser-Doppler perfusion images of Wistar (a1), Wistar + Sham (a2), Wistar + EA (a3), SHR (a4), SHR + Sham (a5), and SHR + EA (a6) group, respectively, acquired at week 16. Black arrow indicates the highest CBF, while white arrow indicates the lowest CBF. (b) Quantitative evaluation of CBF in six groups. Wistar: Wistar rats without any treatment. Wistar + Sham: Wistar rats with stimulation at nonacupoints. Wistar + EA: Wistar rats with stimulation at acupoints. SHR: SHR without any treatment. SHR + Sham: SHR with stimulation at nonacupoints. SHR + EA: SHR with stimulation at acupoints. Data were expressed as mean ± SD from six animals. *P < 0.05 versus Wistar group; ^#^ *P* < 0.05 versus SHR group.

The representative color images of rat CBF acquired at week 16 by Laser Doppler perfusion image system (PeriScan PIM3 System; PERIMED, Stockholm, Sweden) in the six groups are illustrated in Figure 2(a) wherein the different magnitude of CBF is indicated by distinct color, with the red (black arrow in a1) representing the highest CBF and black (white arrow in a4) representing the lowest.  Figure 2(b) is statistical results of CBF in different groups, showing that as compared to Wistar group, CBF in SHR group decreased significantly. Electroacupuncture treatment at acupoints markedly relieved CBF in SHR. In contrast, electroacupuncture treatment at nonacupoints had no significant effect on CBF, although some trend to increase occurred. No significant difference was observed between SHR + Sham and SHR + EA groups either, probably due to the large standard deviation.

### 3.3. Long-Term Stimulation with Electroacupuncture Attenuates the Spatial Learning and Memory Impairment in SHR

**Figure 3:**
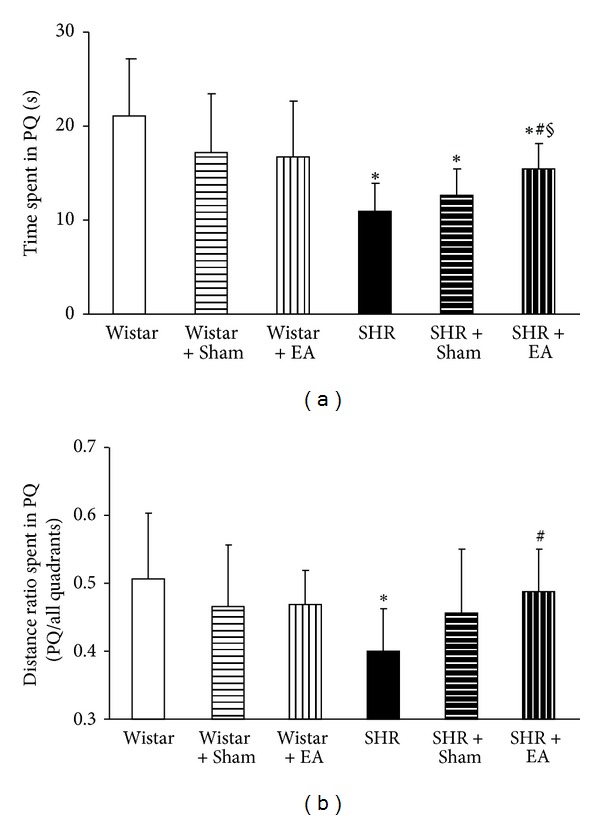
The effect of electroacupuncture on learning and memory capacity of rat. (a) The effect of electroacupuncture on time spent in PQ. (b) The effect of electroacupuncture on distance ratio spent in PQ. Wistar: Wistar rats without any treatment. Wistar + Sham: Wistar rats with stimulation at nonacupoints. Wistar + EA: Wistar rats with stimulation at acupoints. SHR: SHR without any treatment. SHR + Sham: SHR with stimulation at nonacupoints. SHR + EA: SHR with stimulation at acupoints. Data were expressed as mean ± SD from six animals. *P < 0.05 versus Wistar group; ^#^ *P* < 0.05 versus SHR group; ^§^ *P* < 0.05 versus SHR + Sham group.

Figure 3 illustrates the latency and swimming distances assessed by the Morris water maze for rats in different experimental groups. There was no significant difference observed among Wistar group, Wistar + EA group, and Wistar + Sham group in learning and memory potential. Longer latency and swimming distances were observed in SHR group compared to Wistar group. SHR rats that received electroacupuncture treatment displayed a significant improvement in spatial learning and memory impairment as compared with the rats in SHR, exhibiting a smaller mean latency and a shorter mean swimming distance. In comparison to SHR + Sham group, however, rats in SHR + EA displayed an improvement in mean latency but not in mean swimming distance. The impairment of spatial learning and memory was not alleviated by electroacupuncture at non-acupoints.

### 3.4. Long-Term Stimulation with Electroacupuncture Protects the Neuron in CA1 Region of Hippocampus in SHR

**Figure 4:**
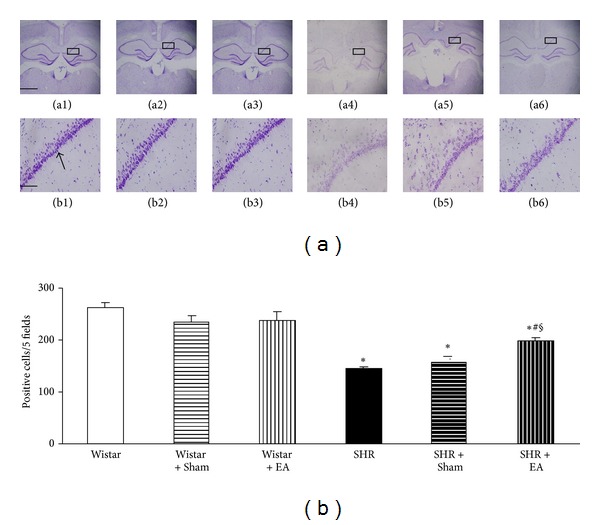
The effect of electroacupuncture on Nissl staining-positive neurons in rat hippocampal CA1 region. (a) Representative Nissl staining images at the end of observation in rat hippocampal CA1 region (arrow) of Wistar (a1), Wistar + Sham (a2), Wistar + EA (a3), SHR (a4), SHR + Sham (a5), and SHR + EA (a6) group, respectively. Bar = 50 *μ*m. b1–b6, high magnification of a1–a6, respectively. Bar = 200 *μ*m. (b) Quantitative evaluation of Nissl staining-positive neurons. Wistar: Wistar rats without any treatment. Wistar + Sham: Wistar rats with stimulation at nonacupoints. Wistar + EA: Wistar rats with stimulation at acupoints. SHR: SHR without any treatment. SHR + Sham: SHR with stimulation at nonacupoints. SHR + EA: SHR with stimulation at acupoint. Data were expressed as mean ± SD from six animals. *P < 0.05 versus Wistar group; ^#^ *P* < 0.05 versus SHR group; ^§^ *P* < 0.05 versus SHR + Sham group.

The number and morphology of neurons in hippocampal CA1 region (arrow) were assessed in different groups at week 16 by Nissl staining, and the results are presented in Figure 4. In Wistar group, the pyramidal cells existed in approximately three to four layers and packed regularly with the Nissl bodies being darkly stained (a1, b1). In contrast, the feature of CA1 region of SHR rats at week 16 was dramatically distinct, characterized by thinning of the cell layers, shrinkage and disintegration of neurons (a4, b4). These morphological changes were attenuated by electroacupuncture at DU20 and ST36 (a6, b6), but not at nonacupoints (a5 and b5).  Figure 4(b) shows a quantitative evaluation of the cell number in CA1 region in various groups. As compared to Wistar rats, the neuron number decreased significantly in CA1 region in SHR and SHR + Sham groups, which was protected by electroacupuncture at DU20 and ST36.

### 3.5. Long-Term Stimulation with Electroacupuncture Increases the Number of Opening Microvessels in Hippocampus of SHR

**Figure 5:**
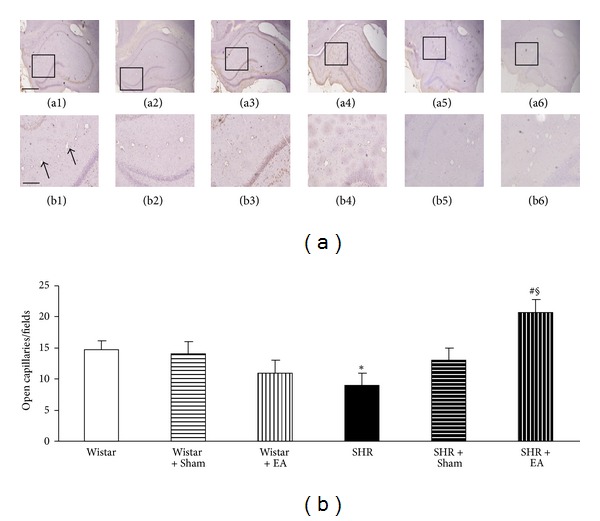
The effect of electroacupuncture on the number of opening microvessels in rat hippocampus. (a) Representative immunohistochemistry images at the end of observation in rat hippocampus of Wistar (a1), Wistar + Sham (a2), Wistar + EA (a3), SHR (a4), SHR + Sham (a5), and SHR + EA (a6) group, respectively. Bar = 50 *μ*m. b1–b6, high magnification of a1–a6, respectively. Arrows indicate opening microvessels. Bar = 200 *μ*m. (b) Quantitative evaluation of CD31-positive opening microvessels. Wistar: Wistar rats without any treatment. Wistar + Sham: Wistar rats with stimulation at nonacupoints. Wistar + EA: Wistar rats with stimulation at acupoints. SHR: SHR without any treatment. SHR + Sham: SHR with stimulation at nonacupoints. SHR + EA: SHR with stimulation at acupoints. Data were expressed as mean ± SD from six animals. *P < 0.05 versus Wistar group; ^#^ *P* < 0.05 versus SHR group; ^§^ *P* < 0.05 versus SHR + Sham group.

To evaluate the number and morphology of microvessels, an immunochemistry staining for CD31 was performed to delineate the vessels (arrows).  Figure 5(a) shows representative images of hippocampal CA1 region stained by immunohistochemisty for CD31 in the six groups. A survey at low power revealed that open microvessels in Wistar group, as well as in Wistar + EA group and Wistar + Sham group, were densely and uniformly distributed in a region between CA1 and dentate gyrus (a1, b1; a2, b2 and a3, b3). Impressively, in SHR and SHR + Sham groups, the density of open microvessels was reduced (a4 and a5), and their distribution became heterogeneous with obviously contractive vasculature and thickening vessel wall (b4 and b5). Compared to SHR and SHR + Sham rats, electroacupuncture at DU20 and ST36 attenuated the alteration in microvessels (a6 and b6), while electroacupuncture at nonacupoints had no effect (a5, b5). A quantitative evaluation of the number of open microvessels confirmed the survey results (Figure 5(b)).

### 3.6. Long-Term Stimulation with Electroacupuncture Reduces the ET-1 but Not NO Content in Brain Tissue of SHR

**Figure 6:**
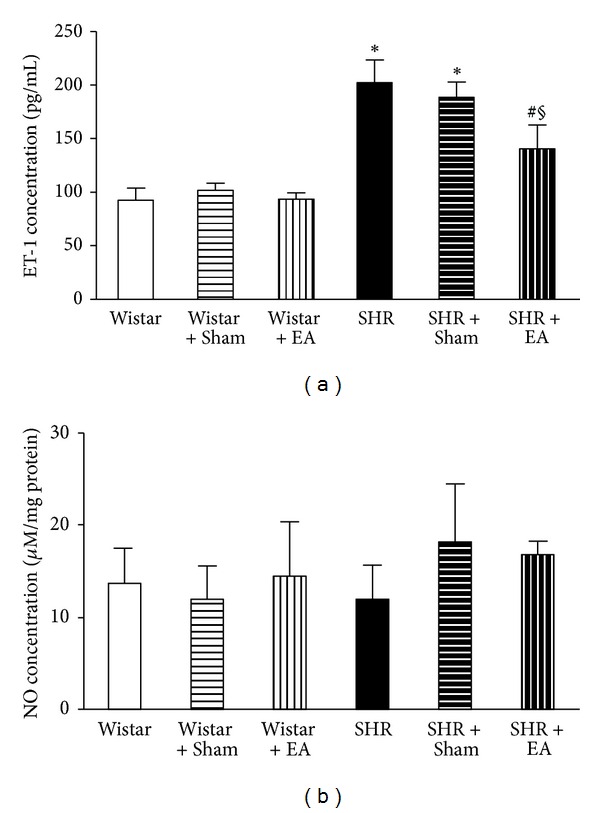
The effect of electroacupuncture on ET-1 and NO concentration in rat brain. (a) The effect of electroacupuncture on ET-1 concentration in rat brain. (b) The effect of electroacupuncture on NO concentration in rat brain. Wistar: Wistar rats without any treatment. Wistar + Sham: Wistar rats with stimulation at nonacupoints. Wistar + EA: Wistar rats with stimulation at acupoints. SHR: SHR without any treatment. SHR + Sham: SHR with stimulation at nonacupoints. SHR + EA: SHR with stimulation at acupoints. Data were expressed as mean ± SD of six animals. *P < 0.05 versus Wistar group; ^#^ *P* < 0.05 versus SHR group; ^§^ *P* < 0.05 versus SHR + Sham group.

The brain content of ET-1 and NO, the two mediators with opposite action on blood pressure, was assessed by ELISA at week 16 in the different groups to explore the mechanism for electroacupuncture effect. As shown in Figure 6(a), there was no difference found in the content of ET-1 in brain homogenate among Wistar group, Wistar + EA group, and Wistar + Sham group. In contrast, ET-1 content increased significantly in SHR and SHR + Sham groups, as compared to Wistar group, which was attenuated by electroacupouncture at DU20 and ST36, but not at nonacupoints. On the other hand, the content of NO did not differ statistically in all the groups studied (Figure 6(b)), indicating that NO does not play a role in augmenting blood pressure in SHR, and is not a mechanism of the electroacupuncture effect.

### 3.7. Long-Term Stimulation with Electroacupuncture Attenuates the AT1R and ETAR but Not AT2R, Expression in Brain Tissue of SHR

**Figure 7:**
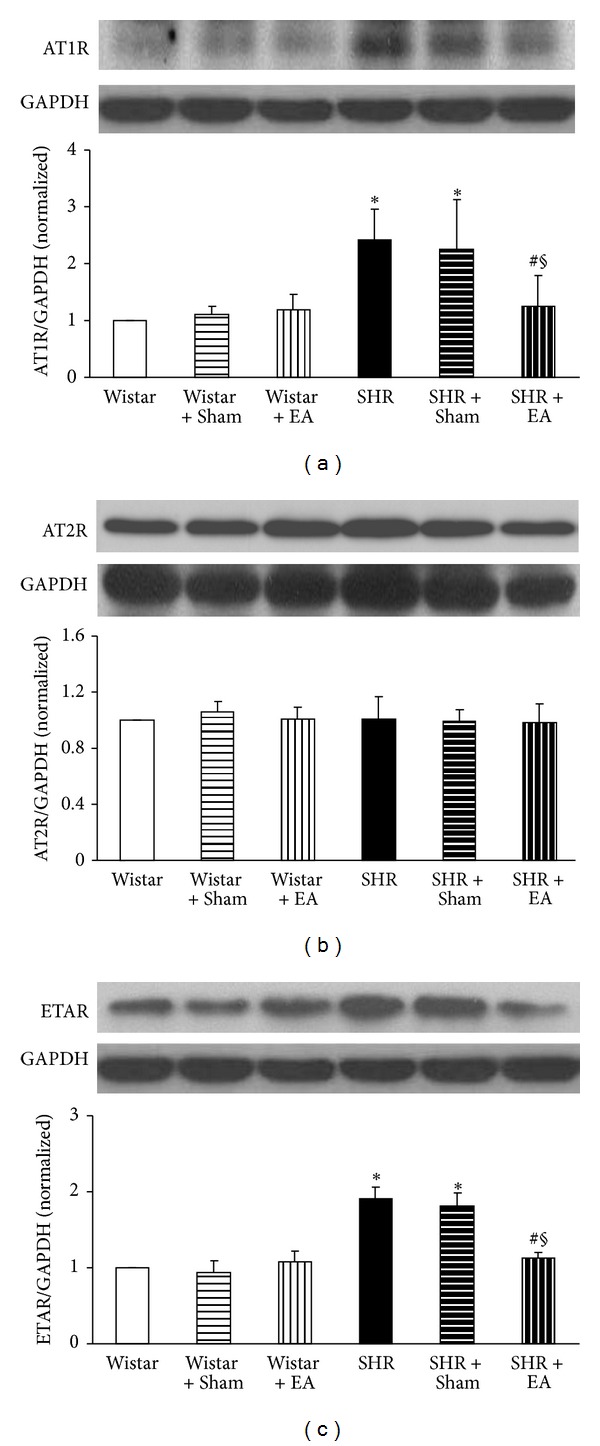
The effect of electroacupuncture on expression of AT1R (a), AT2R (b), and ETAR (c) in rat cerebral tissue. For each protein, the representative Western blot of each group is presented with the respective quantification showing below. Wistar: Wistar rats without any treatment. Wistar + Sham: Wistar rats with stimulation at nonacupoints. Wistar + EA: Wistar rats with stimulation at acupoints. SHR: SHR without any treatment. SHR + Sham: SHR with stimulation at nonacupoints. SHR + EA: SHR with stimulation at acupoints. Data were expressed as mean ± SD from three animals. *P < 0.05 versus Wistar group; ^#^ *P* < 0.05 versus SHR group; ^§^ *P* < 0.05 versus SHR + Sham group.

To elucidate the pathogenesis of hypertension of SHR and the target for electroacupuncture effect observed in the present case, the expression of AT1R, AT2R, and ETAR in brain tissue was determined by Western blot in different conditions at week 16, and the results are presented in Figure 7. AT1R and ETAR expression did not differ obviously among the Wistar group, Wistar + EA group, and Wistar + Sham group, but increased significantly in SHR group, as compared to Wistar group. Electroacupuncture stimulation at acupoint DU20 and ST36 significantly suppressed the increased AT1R and ETAR expression of SHR rats, but had no effect at nonacupoints (Figures 7(a) and 7(c)). In contrast with AT1R and ETAR, there was no obvious difference in AT2R protein level among the six experiment groups (Figure 7(b)). 

### 3.8. Long-Term Stimulation with Electroacupuncture Has No Effects on eNOS and iNOS Expression in Brain Tissue of SHR

**Figure 8:**
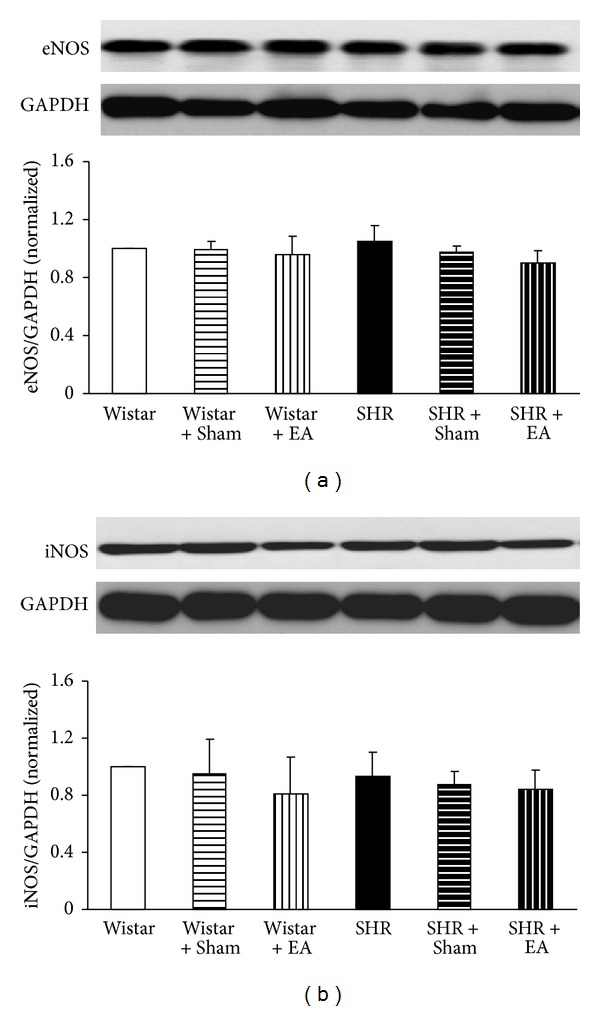
The effect of electroacupuncture on expression of eNOS (a) and iNOS (b) in rat cerebral tissue. For each protein, the representative Western blot of each group is presented with the respective quantification showing below. Wistar: Wistar rats without any treatment. Wistar + Sham: Wistar rats with stimulation at nonacupoints. Wistar + EA: Wistar rats with stimulation at acupoints. SHR: SHR without any treatment. SHR + Sham: SHR with stimulation at nonacupoints. SHR + EA: SHR with stimulation at acupoints. Data were expressed as mean ± SD from three animals.

Similar to AT2R, the expression of eNOS and iNOS proteins in brain tissue did not change significantly among the six experiment groups, as shown in Figures 8(a) and 8(b).

## 4. Discussion

The present study revealed that SHR benefits from the long-term electroacupuncture stimulation at DU20 and ST36 significantly, including relief of hypertension, increase in the number of opening microvessels and cerebral blood flow, attenuation of neuron injury, and restoration of cognitive impairment.

Our preliminary experiments showed that compared to other acupuncture points, such as Yanglingquan (GB34), Hegu (LI4), Quchi (LI11), and Neiguan (PC6), electroacupuncture stimulation at DU20 and ST36 exerts a more apparent antihypertensive effect. Stimulation at ST36 alone was also reported to attenuate hypertension; however, the study regarding its ameliorating effect on cognitive impairment in hypertensive rats is limited [19]. On the other hand, acupuncture stimulation at DU20 is found more effective for improving cognitive impairment in clinic [20]. In the present study, electroacupuncture stimulation at DU20 combined with ST36 relieved hypertension in SHR as well as recovered cognitive impairment.

The morphological changes in the systemic microvasculature are the end result of established hypertension. This alteration may be ascribed to a rarefaction at the capillary level, which plays a significant role in the reduction of CBF induced by hypertension [21, 22]. It has been previously demonstrated that long-term cerebral arteriolar contraction causes the decrease in the number of open microvessels and diminishes the CBF in SHR [23, 24]. The clinical application of calcium channel blockers, diuretics, *β*-receptor blockers, angiotensin converting enzyme inhibitors, and AT1R antagonists can relieve vasospasm and reduce blood pressure [25]. However, these drugs have little effect on CBF. The present study showed that the decreased CBF could be significantly restored by long-term electroacupuncture stimulation therapy at two acupoints DU20 and ST36. In addition, using immunohistochemistry method, we revealed that long-term electroacupuncture could attenuate the reduction in the number of opening capillaries in hippocampus of SHR. The neurons in the hippocampus CA1 region are known to be vulnerable to ischemia and hypoxia. Ischemia and hypoxia induced by rarefaction during hypertension is the major cause responsible for the damage of CA1 hippocampal region neurons and cognitive deficits in SHR [26, 27]. Therefore, ameliorating cerebral vasospasm is anticipated to reduce CA1 region neuron damage and alleviate the cognitive dysfunction. Previous studies showed that MAP increased from 6 weeks [28, 29] and learning and memory dysfunction occurred from 12 weeks in SHR [30]. By virtue of Nissl staining and Morris water maze detection, our work further demonstrated that electroacupuncture at DU20 and ST36 significantly offset hippocampus CA1 neuron lost and learning and memory impairment in SHR, the outcomes that are attributable to the relief of capillary rarefaction.

Existence of an AT1R-ET-1-ETAR pathway in hypertension pathogenesis is well recognized; that is, an increased interaction of AngII with AT1R stimulates the release of ET-1 in endothelial cells that enhances the binding of ET-1 with ETAR, leading to vasoconstriction [31]. Amelioration of AT1R and ETAR expression is thus pivotal for attenuating vasoconstriction and high blood pressure. Our study suggested an implication of AT1R-ET-1-ETAR pathway in alleviating MAP and CBF by electroacupuncture at DU20 and ST36, which suppressed the expression of AT1R and ETAR and reduced the content of ET-1 in SHR. On the other hand, as compared with Wistar rats, no change was observed in the expression of AT2R and the amount of NO. NO is an important molecule that plays a role in a variety of physiological functions, which is synthesized by NO synthase (NOS) [32]. Previous study has reported that therapeutic effects of acupuncture on hypertension are correlated with activation of NOS [9]. In contrast, our results precluded the involvement of AT2R-eNOS/iNOS-NO pathway in the present circumstance, which might be due to the difference in organ and acupuncture sites studied.

 The present study has some limitations. Firstly, the finding of the present study was derived from the observation on the effect of simultaneous application of EA at DU20 and ST36. In a preliminary study, we evaluated the effect of EA on the MAP at 16 weeks by stimulating at either both DU20 and ST36 acupoints or only one of the two. The result showed a more pronounced effect of EA when applied at both acupoints than that at any single one. Since the objective of this study was to explore the mechanisms whereby EA attenuates hypertension, we thus chose a most effective application manner, that is, stimulation at both acupoints. The signaling pathway that mediates the effect of AE on DU20 or ST36 alone is at present unknown, and waits for further study. Secondly, EA is a strategy that combines the acupuncture with electrical stimulation. Researchers have reported that electrical and manual acupuncture stimulation affect glucose homeostasis through different mechanisms [33]. Whether or not the findings of the present study may be extrapolated to manual acupuncture remains to be verified. Finally, mean arterial pressure in SHR + EA group dropped obviously at week 16 compared with that at week 14. What was taking place in mean arterial pressure during the period between the two weeks is not clear. To localize the time point exactly when the effect of EA starts displaying, probably, one or two more time points between week 14 and 16 need to be examined. 

 In conclusion, the long-term electroacupuncture at acupoints DU20 and ST36 relieves the increased MAP and cerebral abnormality in both structure and function in SHR, this beneficial action is most likely mediated via inhibition of AT1R-ETAR-ET-1 pathway. However, two issues remain to be resolved in the future. The first is to confirm that MAP decrease is causative rather than epiphenomenal in the therapeutic effect of acupuncture at acupoints DU20 and ST36 on cerebral protection, and the second is to determine the relation of these findings with other vital pathways, such as endocrine system and meridian system.
