# Corrigendum to “Long-Term Stimulation with Electroacupuncture at DU20 and ST36 Rescues Hippocampal Neuron Through Attenuating Cerebral Blood Flow in Spontaneously Hypertensive Rats”

**DOI:** 10.1155/ecam/9816526

**Published:** 2025-06-06

G. Tian, K. Sun, P. Huang, C. Zhou, H. Yao, Z. Huo, H. Hao, L. Yang, C. Pan, K. He, J. Fan, Z. Li, J. Han, “Long-Term Stimulation with Electroacupuncture at DU20 and ST36 Rescues Hippocampal Neuron Through Attenuating Cerebral Blood Flow in Spontaneously Hypertensive Rats,” *Evidence-Based Complementary and Alternative Medicine* (2013), https://doi.org/10.1155/2013/482947.

In the article, duplication in Figure 1(a) was noted on PubPeer [1], whereby the panel a1 (Wistar) is the same as a3 (Wistar + EA) and panel b1 (Wistar) is the same as b3 (Wistar + EA).

The authors explained that during the experiment, they randomly took three rats in each group for Nissl staining but when uploading, images of Rat 2 in the Wistar + EA group instead of image from Rat 1 in the Wistar group were mistakenly uploaded.  Figure 1 should be corrected as shown below:

The authors apologize for this error.

## Figures and Tables

**Figure 1:**
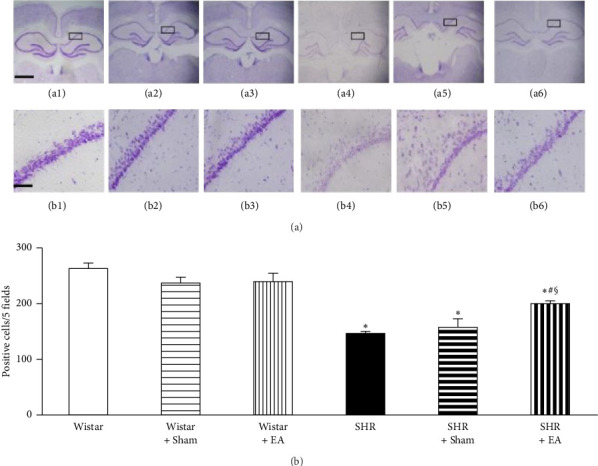
The effect of electroacupuncture on Nissl staining-positive neurons in rat hippocampal CA1 region. (a) Representative Nissl staining images at the end of observation in rat hippocampal CA1 region (arrow) of Wistar (a1), Wistar + sham (a2), Wistar + EA (a3), SHR (a4), SHR + sham (a5), and SHR + EA (a6) group, respectively. Bar = 50 *μ*m. b1–b6, high magnification of a1–a6, respectively. Bar = 200 *μ*m. (b) Quantitative evaluation of Nissl staining-positive neurons. Wistar: Wistar rats without any treatment. Wistar + sham: Wistar rats with stimulation at nonacupoints. Wistar + EA: Wistar rats with stimulation at acupoints. SHR: SHR without any treatment. SHR + sham: SHR with stimulation at nonacupoints. SHR + EA: SHR with stimulation at acupoint. Data were expressed as mean ± SD from six animals. ^∗^*p* < 0.05 versus Wistar group; ^#^*p* < 0.05 versus SHR group; ^§^*p* < 0.05 versus SHR + sham group.
